# Supplementary material for: A Critical View of the Application of the APEX Software (Aqueous Photochemistry of Environmentally-Occurring Xenobiotics) to Predict Photoreaction Kinetics in Surface Freshwaters
Source: Molecules. 2019 Dec 18;25(1):9. doi: 10.3390/molecules25010009 (PMC7017383; doi:10.3390/molecules25010009)
Supplement: Supplementary file 1 [file molecules-25-00009-s001.zip › 3.2.4_gcc-4.4.0.zip!/3.2.4_gcc-4.4.0/3.2.4_gcc-4.4.0/doc/octave/html/interpreter/Manipulating-Structures.html]

Manipulating Structures - Untitled


Next: Processing Data in Structures,
Previous: Creating Structures,
Up: Data Structures


---

#### 6.1.4 Manipulating Structures

Other functions that can manipulate the fields of a structure are given below.

— Built-in Function: **rmfield** (s, f)  
> Remove field f from the structure s. If f is a
> cell array of character strings or a character array, remove the
> named fields.
>
>
>
> **See also:** cellstr, iscellstr, setfield.

— Function File: [k1, ..., v1] = **setfield** (s, k1, v1, ...)  
> Set field members in a structure.
>
> ```
>           oo(1,1).f0 = 1;
>           oo = setfield (oo, {1,2}, "fd", {3}, "b", 6);
>           oo(1,2).fd(3).b == 6
>           => ans = 1
> ```
>
> Note that this function could be written
>
> ```
>           i1 = {1,2}; i2 = "fd"; i3 = {3}; i4 = "b";
>           oo(i1{:}).(i2)(i3{:}).(i4) == 6;
> ```
>
>
>
>
> **See also:** getfield, rmfield, isfield, isstruct, fieldnames, struct.

— Function File: [t, p] = **orderfields** (s1, s2)  
> Return a struct with fields arranged alphabetically or as specified
> by s2 and a corresponding permutation vector.
>
> Given one struct, arrange field names in s1 alphabetically.
>
> Given two structs, arrange field names in s1 as they appear
> in s2. The second argument may also specify the order in
> a permutation vector or a cell array of strings.
>
>
>
> **See also:** getfield, rmfield, isfield, isstruct, fieldnames, struct.

— Built-in Function: **fieldnames** (struct)  
> Return a cell array of strings naming the elements of the structure
> struct. It is an error to call `fieldnames` with an
> argument that is not a structure.

— Built-in Function: **isfield** (expr, name)  
> Return true if the expression expr is a structure and it includes an
> element named name. The first argument must be a structure and
> the second must be a string.

— Function File: [v1, ...] = **getfield** (s, key, ...)  
> Extract fields from a structure. For example
>
> ```
>           ss(1,2).fd(3).b = 5;
>           getfield (ss, {1,2}, "fd", {3}, "b")
>           => ans = 5
> ```
>
> Note that the function call in the previous example is equivalent to
> the expression
>
> ```
>           i1 = {1,2}; i2 = "fd"; i3 = {3}; i4= "b";
>           ss(i1{:}).(i2)(i3{:}).(i4)
> ```
>
>
>
>
> **See also:** setfield, rmfield, isfield, isstruct, fieldnames, struct.

— Function File: **substruct** (type, subs, ...)  
> Create a subscript structure for use with `subsref` or
> `subsasgn`.
>
>
>
> **See also:** subsref, subsasgn.
